# Optimization of growth and production parameters of walnut (*Juglans regia*) saplings with response surface methodology

**DOI:** 10.1038/s41598-018-28345-6

**Published:** 2018-07-03

**Authors:** Dagang Song, Kaiwen Pan, Aiping Zhang, Xiaogang Wu, Akash Tariq, Wenkai Chen, Zilong Li, Feng Sun, Xiaoming Sun, Olusanya Abiodun Olatunji, Lin Zhang

**Affiliations:** 1 Key Laboratory of Mountain Ecological Restoration and Bioresource Utilization & Ecological Restoration Biodiversity Conservation Key Laboratory of Sichuan Province, Chengdu Institute of Biology, Chinese Academy of Sciences, Chengdu, 610041 China; 2 University of Chinese Academy of Sciences, Beijing, 10049 China

## Abstract

Straw mulching is an effective conservation tillage tool that utilizes waste resources and reduces environmental pollution. To determine the optimal levels of quality, placement and quantity of straw mulching, we performed a field experiment that used the Box–Behnken design combined with response surface methodology. The treatments designed for walnut saplings (*Juglans regia*) considered three independent variables: quality, placement, and quantity of straw mulching. Tree height of walnut saplings (THW) and net photosynthesis rate (NPR) were used as the response variables in a full, quadratic polynomial model. Analysis of variance (ANOVA) results showed that the selected models were significant (*P* < 0.05), expressing ideal relationships between the independent and dependent variables (R^2^ ≥ 0.9225). The optimum conditions for the THW and NPR responses were determined to be a straw mulching quality which mixed rice and rapeseed straws, a straw mulching placement which covered the entire soil surface of experimental plots, and a straw mulching quantity applied as 3 kg/m^2^ (i.e., the low level). This optimized scheme of straw mulching combinations offers an alternate choice for optimizing the growth and potential yield of walnut saplings, but practical field experiments should also be carried out to obtain more site-specific results.

## Introduction

Common walnut (*Juglans regia L*.) is tree species of economic importance well known throughout the world and one of the main fruit trees planted in China^1^. Since 1998, walnut has become widely cultivated in the hills and mountains of China during the country’s implementation of its “Grain for Green Project”. As such, walnut production in China has risen rapidly during 2001–2012, accounting for 55.25% of the world’s total annual output in 2012 (FAO, 2013). Walnut plantations not only produce considerable economic benefits but also provide important ecological services^2^. In seeking to promote the tree growth and fruit yield of walnut plantation, most studies have focused on weed control^3^, fertilization^4^, irrigation^5^, intercropping^6–9^, and thinning^10^ interventions. By contrast, there are surprisingly few reports of how conservation tillage, such as the activity of straw mulching, affects the tree growth and yield of walnut saplings in plantations.

Straw resources are abundant in China. It has 700 million tons of crop straw available every year, of which rice straw and rape straw amount to 201.97 and 106.10 million tons, respectively. Yet the majority of this straw is often burned in the field because of ineffective methods to use it, which represents a great waste of resources that also causes air pollution^11^. To reduce straw burning, and to enhance the utilization efficiency of crop straw in particular, it is imperative that effective ways for using this available straw are investigated in those areas where walnut, rice, and rape crops are planted. To avoid the huge costs of transporting large amounts of straw over long distances around the country, we should take advantage of recycling crop straw that is near walnut orchards. Such an initiative would attract the attention and interest of the government, local people and ecologists.

Recycling straw resources is a use that can partially substitute for chemical fertilizer, thus providing an important avenue towards maintaining environment-friendly and sustainable agriculture^12^. Straw mulching is considered a key criterion of sustainable agricultural management, one capable of improving crop photosynthesis and growth^13–15^, altering soil physical properties and increasing soil nutrients^16^, as well as augmenting crop yields^17^. However, most studies of straw mulching have mainly focused on its effects on crop fields rather than on tree orchards, much less on economically important orchards. The traditional management practices for walnut orchards, such as clean tillage, often result in serious soil erosion and fertility declines, thereby reducing the growth and yield of the walnut trees^18^. Hence, it is worthwhile and timely to consider using straw mulching to improve the sustainable development of walnut orchards.

Suitable straw mulching material can produce notable economic and ecological benefits, such as conserving local soil and water, improving soil chemical and physical properties, controlling weeds, and promoting woody plant growth^19–21^. When applied in a suitable placement around the target tree trunk, straw mulching can change the micro-environmental factors available for tree growth, depending on the mulch properties/types, quantity, and its interaction with the soil^22^. When considering the influences from canopy crown shading, it seems reasonable to design the mulching horizontal placement according to the radius of the canopy crown. Moreover, applying too much, or too little straw mulch, may negatively affect plant maturity, height and soil nitrate production^14,23^. Therefore, determining the appropriate quantity of straw mulch to apply *in situ* is an essential step towards the sustainable management of walnut orchards.

Complex interactions can occur, however, among straw mulching quantity, its type, and the placement on the soil for targeted tree growth. Clearly, it is far from ideal to assess the effects of treatment combinations on an orchard ecosystem through conventional multi-factorial experiments, given their high expenditures in terms of time, manpower and money. Alternatively, response surface methodology (RSM) offers an efficient way to estimate the effects of individual factors and their interactions^24^. In RSM a limited number of experiments are used to evaluate the interactions of possible factors^25^. In the present paper, we chose the straw of main cereal and oil crops in southern China to serve as the experimental material. Our study objective was to derive the optimal combination of straw mulching type, placement and quantity based on a Box–Behnken design (BBD) for supporting straw mulch utilization in walnut orchard management.

## Results

### Influencing parameters

**Figure 1:**
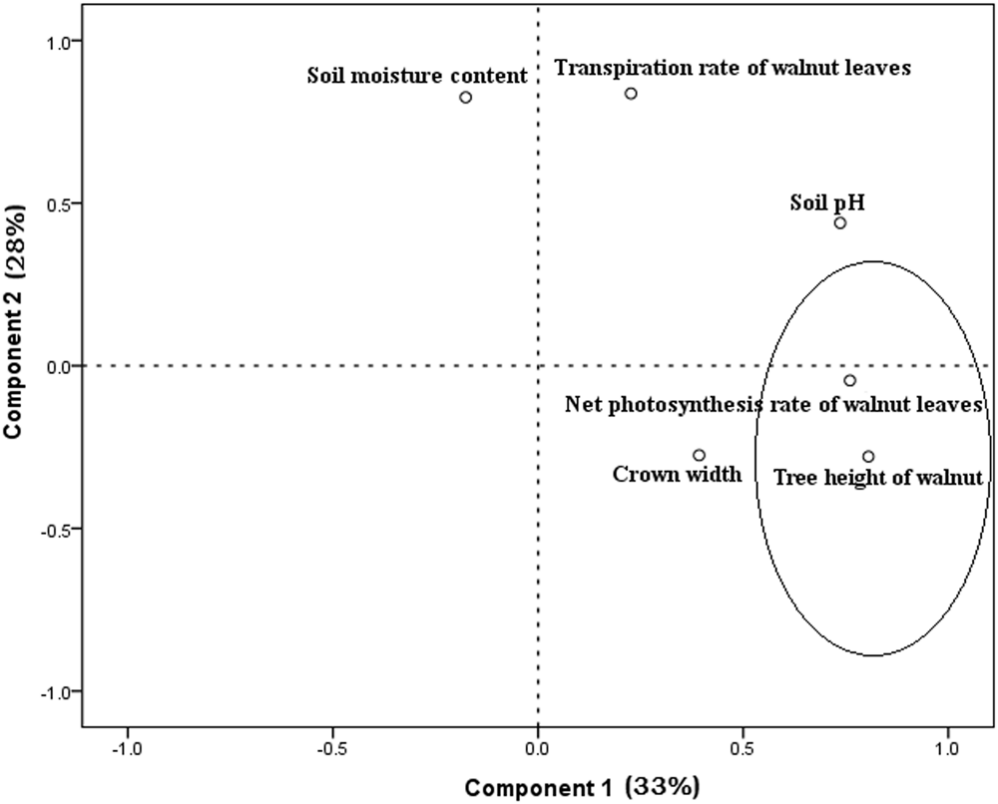
Principal component analysis of walnut saplings growth parameters.

The two extracted components represented the most complete information given their higher scores (Table 1). Component 1 had a strong positive correlation with tree height, leaf net photosynthesis rate and soil pH, while component 2 had a strong positive correlation with both leaf transpiration rate and soil moisture. Component 1 (33%) better explained the total analysis results than did component 2 (28%) for the parameters of tree growth. In component 1, the absolute value of tree height and leaf net photosynthesis rate was large, and the distance between each other was small, so it could represent the actual growth of walnut tree (Fig. 1). Tree height and leaf net photosynthesis rate were selected as the main parameters for the growth of walnut saplings according to the components’ core coefficient matrix.

**Table 1:** Component score coefficient matrix.

	Component 1	Component 2
Tree height of walnut	0.402	−0.161
Net photosynthesis rate of walnut leaves	0.379	−0.026
Crown width	0.196	−0.159
Soil pH	0.367	0.254
Soil moisture content	−0.088	0.477
Transpiration rate of walnut leaves	0.113	0.484

### Response surfaces analysis and contour for tree height

The heights of walnut saplings had a range of 1.5–3.55 m. The linear effect of mulching placement (*P* < 0.01), the interaction effects between the straw mulching quality and straw mulching quantity (*P* < 0.01), and that between the straw mulching placement and straw mulching quantity (*P* < 0.05), and the quadratic effect of straw mulching quality (*P* < 0.01), were all key factors that influenced the tree height of walnut saplings (THW) (Table 2).

**Table 2:** ANOVA for response of tree height and net photosynthesis rate.

Resource	Y_1_ tree height					Y_2_ net photosynthesis rate				
	DF	Sum of squares	Mean square	F-value	Coefficient estimate	DF	Sum of squares	Mean square	F-value	Coefficient estimate
Model	9	6.59	0.73	12.33	3.10**	9	5.14	0.57	9.26	5.25**
X_1_	1	0.00	0.00	0.01	−0.01	1	0.31	0.31	5.06	0.20
X_2_	1	1.36	1.36	22.93	−0.41**	1	0.37	0.37	5.93	−0.21*
X_3_	1	0.20	0.20	3.29	−0.16	1	2.28	2.28	36.97	−0.53**
X_1_X_2_	1	0.33	0.33	5.57	0.29	1	0.40	0.40	6.44	0.31*
X_1_X_3_	1	2.40	2.40	40.46	−0.78**	1	0.79	0.79	12.85	−0.45**
X_2_X_3_	1	0.60	0.60	10.12	−0.39*	1	0.05	0.05	0.82	0.11
X_1_^2^	1	1.64	1.64	27.70	−0.63**	1	0.00	0.00	0.06	−0.029
X_2_^2^	1	0.05	0.05	0.90	0.11	1	0.64	0.64	10.32	0.39*
X_3_^2^	1	0.01	0.01	0.18	−0.050	1	0.26	0.26	4.23	0.25
Residual	7	0.42	0.06			7	0.43	0.06		
Lack of fit	3	0.42	0.14			3	0.43	0.14		
Pure error	4	0.00	0.00			4	0.00	0.00		
Cor total	16	7.00				16	5.57			

X_1_ straw mulching quality, X_2_ straw mulching placement, X_3_ straw mulching quantity; Tree height (R^2^ = 0.9407, Adeq. Precision = 12.407, Std.dev. = 0.24, C.V. = 8.59, Mean = 2.84); Net photosynthesis rate (R^2^ = 0.9225, Adeq. Precision = 10.811, Std.dev. = 0.25, C.V. = 4.48, Mean = 5.54).

*Significant at 5% (*P* < 0.05); ** Significant at 1% (*P* < 0.01).

**Figure 2:**
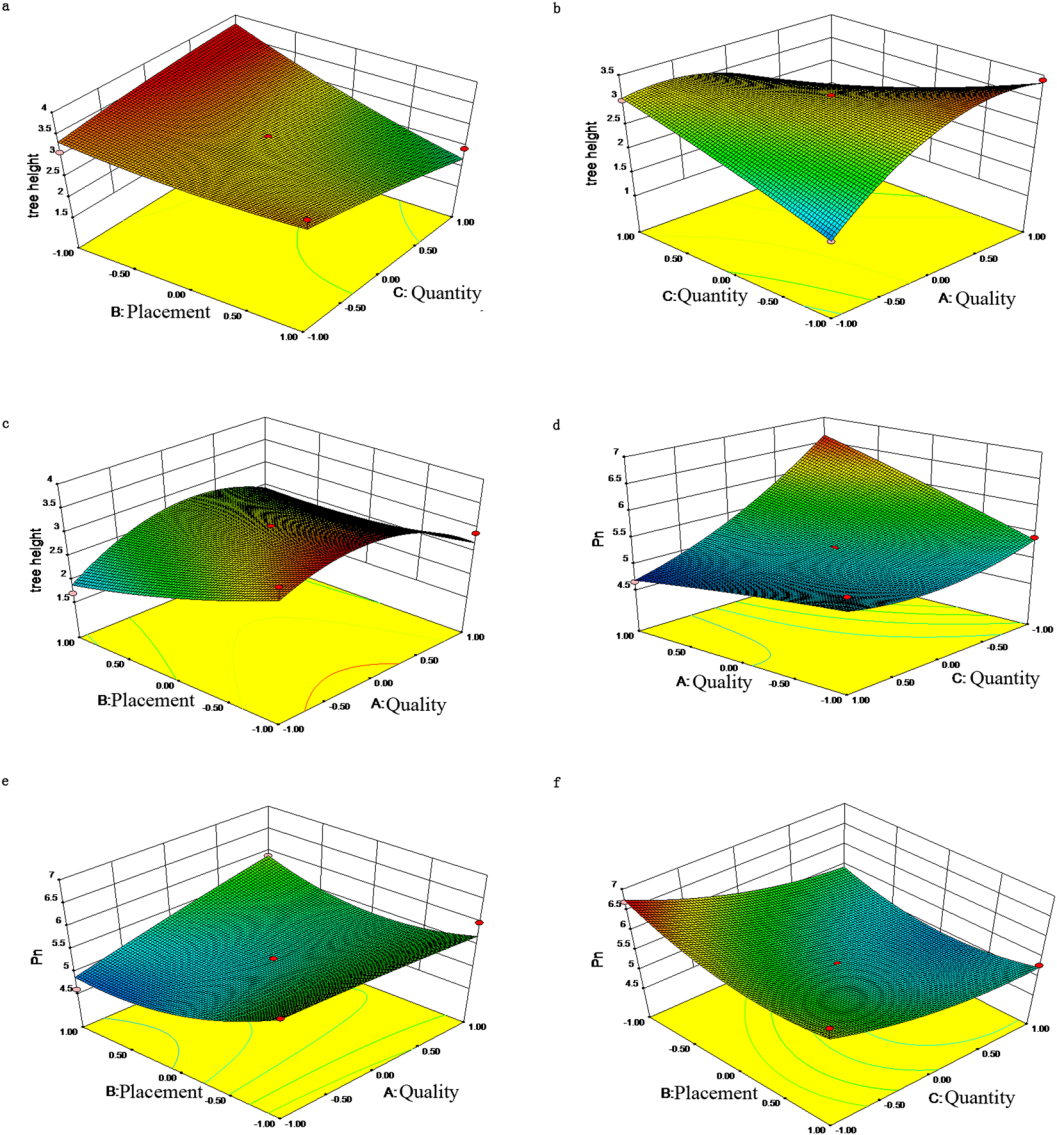
Response surfaces showing the effect of quality, placement, and quantity on height and P_n_ onto walnut saplings.

The placement of straw mulching around the tree trunk had significant negative linear effects on tree height (Fig. 2a). The 3D response surface graphs also showed that tree height decreased as this placement was increased. Increases in both independent variables, including straw mulching quantity and straw mulching quality, led to an improvement in the THW (Fig. 2b). The response surfaces revealed that the THW was raised by an associated increase of straw mulching quantity for all straw mulching quality levels, while an increase in the straw mulching quality level led to THW’s improvement at all straw mulching placement levels. At low levels of straw mulching quality (ranging from the rice to rapeseed qualities), the THW was enhanced, with a sharp slope driven by decreasing straw mulching placement from all straw mulching qualities, whereas a decrease in the straw mulching placement levels did not have a positive effect on THW from the rapeseed quality to the mixed quality under all straw mulching placements (Fig. 2c).

The coefficient of determination (R^2^ = 0.9407) from the analysis of variance (ANOVA) indicated just 5.93% of the total variation was unexplained by the fitted quadratic regression model; hence, it effectively represented the relationships among the selected variables. It is generally considered that an adequacy precision value >4 is desirable; in this study the adequacy precision was 12.4, indicating this model may be used to navigate the design space. A relatively lower value of the coefficient of variation (CV = 8.59%) indicated that the variation in the mean value was acceptable and satisfactory according to its acceptable range of 0.5%–13.5%^26^. The overall second order polynomial equation for THW has the following expression (Eq. 1):

1 $$\begin{array}{rcl}{{\rm{Y}}}_{{\rm{1}}} & = & {\rm{3.10}}-{\rm{0}}{{\rm{.00625X}}}_{{\rm{1}}}-{\rm{0}}{{\rm{.41X}}}_{{\rm{2}}}-{\rm{0}}{{\rm{.16X}}}_{{\rm{3}}}+{\rm{0}}{{\rm{.29X}}}_{{\rm{1}}}{{\rm{X}}}_{{\rm{2}}}\\  &  & -\,{\rm{0}}{{\rm{.78X}}}_{{\rm{1}}}{{\rm{X}}}_{{\rm{3}}}-{\rm{0}}{{\rm{.39X}}}_{{\rm{2}}}{{\rm{X}}}_{{\rm{3}}}-{\rm{0}}{{{\rm{.63X}}}_{{\rm{1}}}}^{{\rm{2}}}+{\rm{0}}{{{\rm{.11X}}}_{{\rm{2}}}}^{{\rm{2}}}-{\rm{0}}{{{\rm{.050X}}}_{{\rm{3}}}}^{{\rm{2}}}\end{array}$$

### Response surface analysis for net photosynthesis rate

The net photosynthesis rate (NPR) of the walnut saplings leaves ranged from 4.59 to 6.7 μmol·m^−2^·s^−1^ throughout the experimental period. Both straw mulching placement (*P* < 0.05) and straw mulching quantity (*P* < 0.01) were negatively correlated with NPR. However, the interaction between the straw mulching quality and the straw mulching placement was positive (*P* < 0.05), whereas that between the straw mulching quality and the straw mulching quantity was negative (*P* < 0.01), and quadratic effect of straw mulching placement was significant (*P* < 0.05).

For all straw mulching qualities examined, the negative effects of straw mulching quantity on NPR increased with greater amounts of mulching (Fig. 2d). However, the quality of straw mulching had almost no effect on NPR under an increasing straw mulching quantity. NPR was improved by increasing the straw mulching quality level at all straw mulching placements (Fig. 2e). Maximum values of NPR, with respect to the placement-quality effect, were obtained by high levels of straw mulching placement as well as by the quality (Fig. 2e); however, NPR was reduced by increasing the straw mulching placement over all quantities of straw mulching (Fig. 2f). The maximum NPR was attained when the lowest straw mulching placement was combined with the lowest straw mulching quantity, whereas its minimum value was observed at the highest straw mulching placement with the lowest straw mulching quantity (Fig. 2f).

The coefficient of determination (R^2^) was 0.9225, which indicated a good fit between the measured and the predicted net photosynthesis rate of walnut saplings leaves (Table 2). The adequacy precision of 10.8 for the Y_2_ response indicated sufficient model discrimination. In addition to this, a low value of the coefficient of variation (CV = 4.48%) denoted good accuracy and reliability of the experiments. Significant terms related to NPR could thus be expressed as follows (Eq. 2):

2 $$\begin{array}{rcl}{{\rm{Y}}}_{{\rm{2}}} & = & {\rm{5.25}}+{\rm{0}}{{\rm{.20X}}}_{{\rm{1}}}-{\rm{0}}{{\rm{.21X}}}_{{\rm{2}}}-{\rm{0}}{{\rm{.53X}}}_{{\rm{3}}}+{\rm{0}}{{\rm{.31X}}}_{{\rm{1}}}{{\rm{X}}}_{{\rm{2}}}\\  &  & -\,{\rm{0}}{{\rm{.45X}}}_{{\rm{1}}}{{\rm{X}}}_{{\rm{3}}}+{\rm{0}}{{\rm{.11X}}}_{{\rm{2}}}{{\rm{X}}}_{{\rm{3}}}-{\rm{0}}{{{\rm{.029X}}}_{{\rm{1}}}}^{{\rm{2}}}+{\rm{0}}{{{\rm{.39X}}}_{{\rm{2}}}}^{{\rm{2}}}+{\rm{0}}{{{\rm{.25X}}}_{{\rm{3}}}}^{{\rm{2}}}{\rm{.}}\end{array}$$

## Discussion

After establishment, walnut sapling height is a measure of its average growth rate^27^. This is one of the important parameters for depicting the success/failure of straw mulching treatments with a view towards increasing fruit yield.

The walnut saplings mainly depend on their root systems to absorb the water needed from the soil, and to transfer water to their organs for height growth. Straw mulching has an obvious water retention effect, especially in dry season, in that it can provide more water for root system uptake, and thus is more conducive to the growth of walnut saplings, especially for early increases in height. This can be explained by the genetic characteristics of a typical walnut sapling: most of its roots are located within the radius of its crown^28^, which is consistent with the straw mulching placement, n (crown-width radius). As such, other straw mulching placements, such as 1.5n or all n, which exceeded the root distribution, could not have a significant impact on the growth of walnut saplings.

Straw mulching quantity also had a significant effect on walnut sapling growth. As the straw mulching quantity increased from 3 kg to 9 kg, the walnut sapling height increased from 1.5 m to 3 m. This augmentation was due to the increased straw quantity, likely providing a more indirect C source that improved the nutritional status of the soil^29^, which would have been favourable for the growth of walnut saplings. In addition, the soil water content was positively correlated with the quantity of straw mulching, and also increased with the increasing of straw mulch quantity^30^. Therefore, increasing the straw mulch quantity likely helps to increase the soil water content, thereby better providing sufficient water for the organs of the walnut saplings via its the root system. For this reason, it was beneficial to the growth of walnut saplings.

The straw mulching qualities had different effects on walnut sapling height, which may be explained by differences in the respective straw components and their decomposition. The carbon nitrogen ratio (C: N) is an important factor affecting the decomposition of straw. Generally, degradation is more difficult, or slowed, when the straw C: N is higher. The C: N of rapeseed straw (100) is much higher than that of rice straw (60)^31,32^. Thus, rape straw degrades more slowly than does rice straw; so, rape straw exerts a better function with respect to soil water retention and soil erosion prevention. In addition, the mixed quality may have provided a stable moisture content and suitable soil texture that favours unrestricted expanded root growth, and thus subsequent increased rates of nutrient absorption^33^.

The yield of crops is mainly produced by photosynthesis. The fundamental way to increase crop yields is to improve the photosynthetic performance of plants by adopting proper cultivation measures (i.e., to maximize photosynthetic capacity). The net photosynthetic rate is thus an important index which reflects plant productivity, and which can be used as an important parameter to estimate crop yield.

Maximum NPR was achieved when a low straw mulching quantity (3 kg/m^2^) was applied at the highest straw mulching quality level (mixed quality) (Fig. 2d). Because the quantity of straw mulching typically used exceeds the maximum carrying capacity, the root respiration of crops becomes weakened and the release of harmful methane gas is increased, which is not conducive to the growth of crops, weakening the overall leaf photosynthetic capacity^34^. Compared with the mulch of rice straw, that of rapeseed straw or the mixed quality could significantly improve its reflectance and ground heat retention, which should enhance the photosynthetic capacity of leaves in the lower canopy of saplings. This is because the colour of rapeseed straw or the mixed quality is whiter than that of rice straw. Sagawa’s studies^35,36^ have shown that the photosynthetic rate increased with a greater coverage placement because this provided more reflected light, which led to an increase in the photosynthetic rate of middle and lower leaves^23^. However, Pan *et al*.^37^ found that with a decreased coverage placement, bamboo’s photosynthetic ability increased, so that the net photosynthetic rate peaked under a mulching placement (i.e., 3-m × 3-m plot). Our results reconcile these two prior viewpoints in a new synthesis: namely, a proper placement of straw mulch could improve the photosynthetic productivity of plants by creating more suitable habitat for them, such as by conserving soil and water, improving soil chemical and physical properties, and controlling weeds, to thereby promote walnut saplings’ growth.

Before the walnut bears fruit, its saplings’ height and photosynthetic rates are important indicators of tree growth and potential yield^27^. Nevertheless, it is difficult to optimize the two responses at the same condition due to the fact that the regions of interest in each factor are different. Thus, to arrive at a compromise for these two responses, desirability functions were applied^38^.

**Figure 3:**
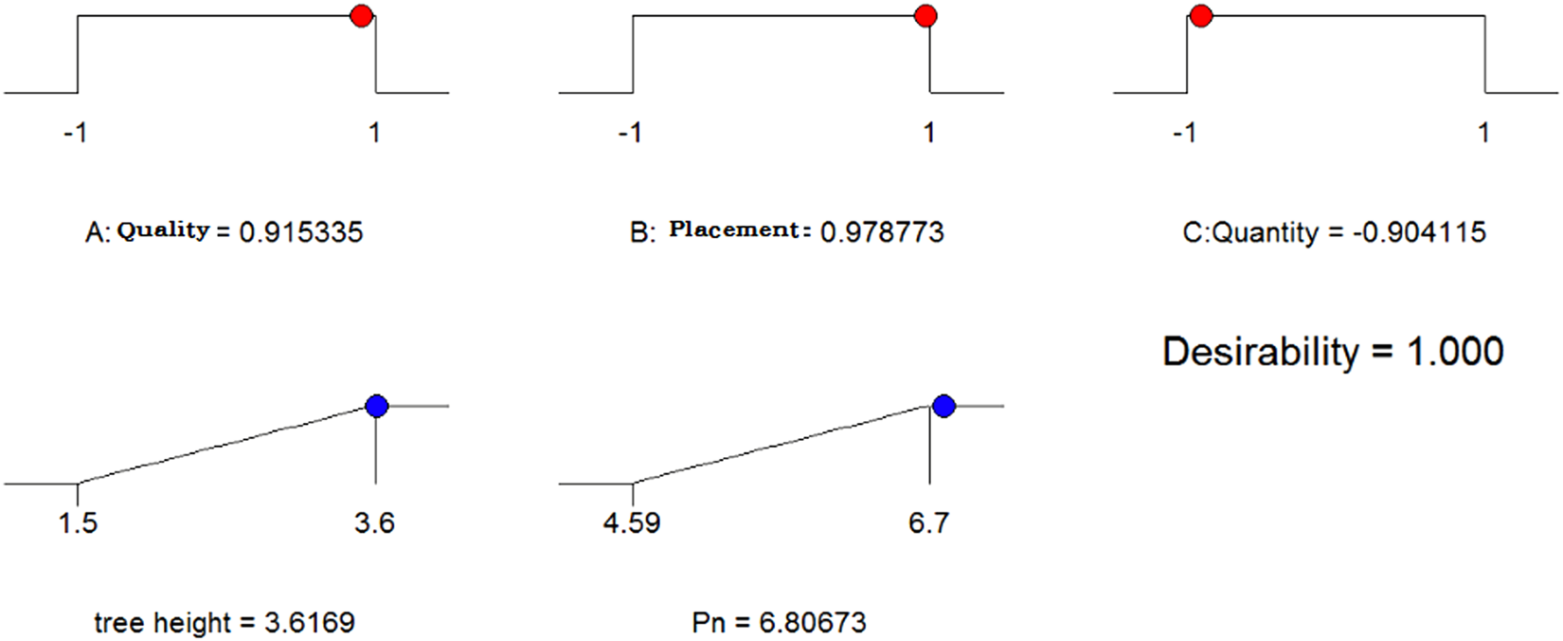
Optimized process condition.

Figure 3 shows the plots of predicted against actual values for tree height and P_n_. Evidently there was good correspondence between both values. The predicted optimal values of the independent variables were as follows: X_1_(A) = 0.915, X_2_(B) = 0.978 and X_3_(C) = −0.904, to achieve the maximum THW and NPR values as shown in Fig. 3 with the overall desirability of 1. Summarized in Table 3 are the optimization results for the THW and NPR values for the straw mulching effect obtained by examining the response curves. These results of 3.43 m and 6.32 μmol·m^−2^·s^−1^ closely agreed with those of 3.61 m and 6.80 μmol·m^−2^·s^−1^ for THW and NPR, respectively, that came from the optimization analysis using the desirability function^39^. This indicated that BBD in combination with the desirability functions could be effectively used to optimize THW and NPR for a different straw mulching quality, placement and quantity. Moreover, the predicted and experimental THW and NPR values were significant higher than the control. This result indicated that straw mulching had positive effects on the growth of walnut saplings. Finally, we obtained the optimal parameters of THW and NPR as follows: mixed quality (0.915), all n (0.978), and 3 kg/m^2^ (−0.904) according to BBD.

**Table 3:** Model Validation.

Quality	Placement	Quantity	Tree height (m)			P_n_ (μmol·m^−2^·s^−1^)		
			Predicted	Experimental	CK	Predicted	Experimental	CK
0.915	0.978	−0.904	3.616	3.43	2.7	6.806	6.32	2.82

Based on these results, we conclude that this optimized straw mulching can benefit sapling growth, thereby reducing the time to first fruit set, which should increase the lifetime yield of walnut trees. Maybe the ranges were not as accurate as they could have been (R^2^ = 0.9407 for tree height; and R^2^ = 0.9225 for net photosynthesis rate); or, perhaps, they require further refinement of the simulation experiments. Nonetheless, the two models were significant, and the optimum conditions of the three parameters are thus a useful reference for future work.

## Methods

### Study site

The field experiment was conducted in Langzhong (31°57′82″N, 105°96′65″E; 712.5 m above sea level), Sichuan Province, south western China, from July to October 2016. The area is part of an agroecosystem and has a humid mid-subtropical monsoon climate, with an average annual precipitation of 1033.9 mm and annual temperature of 18.7 °C. This site has purple soil, classified as Pup-Orthic Entisol in the Chinese Soil Taxonomy (CST) and Entisol in the USDA Soil Taxonomy^40^. The specific soil used in this study was a loam soil with the following nutrient profile (0–15 cm depth): total nitrogen (2.4 g·kg^−1^), available phosphorus (0.96 g·kg^−1^), available potassium (86.57 m g·kg^−1^) and total carbon (5.95 g·kg^−1^)^41^. The walnut sapling (*Juglans regia*) plantation covered a 30-m × 90-m area, with a southerly slope of c. 2.5 degrees. The walnut saplings were planted in April 2010 using 3-m × 3-m spacing, and then grafted in May 2015.

### Experimental design

We used the Box–Behnken design to evaluate the effects of straw mulching^42^. The independent variables included the mulching quality (X_1_), mulching placement (X_2_) and mulching quantity (X_3_) each with low, middle and high levels. The dependent variables were tree height (THW) (Y_1_) and net photosynthesis rate (NPR) (Y_2_) (Table 4). Each independent variable was coded as −1, 0 and +1, respectively, as illustrated in Table 4. The ranges of each parameter were determined according to previous research results^43–45^. Three replications for each dependent parameter were evaluated. The following equation was used to estimate the code values:

3 $${{\rm{X}}}_{{\rm{i}}}=\frac{{{\rm{x}}}_{{\rm{i}}}-{{\rm{x}}}_{0}}{{{\rm{\Delta }}x}_{{\rm{i}}}}$$

Where X_i_ is the dimensionless coded value of the *i-*th variable, x_i_ is the real value of the *i-*th variable, x_0_ is the real value of the *i-*th variable at the centre-point, and Δx_i_ is the step-change value^46,47^.

**Table 4:** Experimental range and level of independent variables

Variables	levels		
	−1	0	+ 1
**Independent variables**			
X_1_ = straw mulching quality	Rice straw	Rapeseed straw	Mixed rice straw and rapeseed straw
X_2_ = straw mulching placement(m)	n	1.5n	all n
X_3_ = straw mulching quantity (kg/m^2^)	3	6	9
Dependent variables			
Y_1_ = Tree height (m)			
Y_2_ = Net photosynthesis rate (μmol·m^−2^·s^−1^)			

n = mean radius of crown width, all n: coverage the whole plots; mixed quality: equal quality mixing with 1:1.

There was a total of 17 combinations, based on three levels and three variables, including five replicates at the centre-point to evaluate the experimental error. All 17 combinations were subjected to random permutations (Table 5). Theoretically, the centre-point of the Box–Behnken design needs to be replicated at least three times, whereas the other treatments do not require such replication^46^. However, given that the variability in field experiments is generally greater than that in laboratory studies, we replicated the centre-point treatment (0, 0 and 0) five times, and the other straw mulching treatment three times each. The experiment had one additional treatment that served as the control: it had no straw mulching, with three replicates. The multiple regression analysis can be expressed by a second-order polynomial model:

4 $${\rm{Y}}={{\rm{\beta }}}_{0}+{\sum }_{{\rm{i}}=1}^{3}{{\rm{\beta }}}_{{\rm{i}}}{{\rm{X}}}_{{\rm{i}}}+{\sum }_{{\rm{i}}=1}^{3}{{\rm{\beta }}}_{{\rm{ii}}}{{\rm{X}}}_{{\rm{i}}}^{2}+{\sum }_{{\rm{i}}=1}^{2}{\sum }_{{\rm{j}}={\rm{i}}-1}^{3}{{\rm{\beta }}}_{{\rm{ij}}}{{\rm{X}}}_{{\rm{i}}}{{\rm{X}}}_{{\rm{j}}}$$

Where Y is the predicted response by the model (i.e., tree height or net photosynthesis rate) and X_i_ and X_j_ are the independent variables, and β_0_, β_i_, β_ii_ and β_ij_ are the regression coefficients of the fitted model. The validity of the predicted model was verified by ANOVA, the second-order model quality assessed by the determination coefficient (R^2^), and the analysis was carried out with a Fisher’s F-test and probability value (with 95% confidence limits or intervals constructed around the mean or regression coefficients). Finally, the optimal values of the tested variables were obtained by analysing the plotted surface curves.

**Table 5:** Experimental design matrix and results.

Run order	Independent variables			Dependent variables	
	X_1_	X_2_	X_3_	Y_1_	Y_2_
1	0	1	1	2.45	5.3
2	1	1	0	2.2	5.9
3	0	0	0	3.1	5.25
4	0	−1	1	3.6	5.2
5	−1	0	1	3	5.43
6	−1	−1	0	3.55	5.95
7	0	0	0	3.1	5.25
8	−1	0	−1	1.8	5.4
9	−1	1	0	1.7	4.59
10	0	1	−1	3.5	6.35
11	0	0	0	3.1	5.25
12	1	0	−1	3.4	6.4
13	0	−1	−1	3.1	6.7
14	0	0	0	3.1	5.25
15	1	0	1	1.5	4.65
16	1	−1	0	2.9	6
17	0	0	0	3.1	5.25

### Straw mulching treatments

The field experiment included three mulching treatments: rice straw, rape straw, and mixed straw mulching. The straw of different types was directly used to cover the soil surface of the walnut orchard on 15 June 2016.

### Straw material

Rice straw and rapeseed straw was collected from Langzhong in Sichuan Province, China, and these samples were allowed to dry naturally. Stalk samples were then stored in a storage room with good ventilation. The basic chemical characterization of the two straw materials is shown in Table 6. The samples were tested according to the standards in the “National Renewable Energy Laboratory-Determination of the Cellulose and Lignin Content in the Biomass Samples” (/TP-510-42618, NREI), “ASTM E777-08 Standard Test Method for Carbon and Hydrogen in the Analysis Sample of refuse-Derived Fuel”.

**Table 6:** Chemical characterization of the two straw materials.

Material	Total C (%)	Total N (%)	Cellulose (%)	Hemicelluloses (%)	Lignin (%)
Rice straw	38.32	0.62	29.53	15.59	29.68
Rapeseed straw	46.21	0.45	52.36	20.56	11.63

### Measurements

#### Soil sampling and analysis

Soil samples were collected on 19 October 2016. In each plot, a total of five soil cores (5 cm in diameter) were obtained by using the five-spot method with a 20-cm soil depth, and combined to form one composite sample per plot location. Each composite sample was placed in a plastic bag and transported to the laboratory. There, the soil samples were air-dried at room temperature and sieved by 2 mm before determining the concentrations of soil properties, namely soil pH and soil moisture content. The latter was estimated gravimetrically, by oven drying (at 105 °C for 24 h) 20 g of each field composite soil sample. Soil pH was estimated at in a 1:2.5 soil/CaCl_2_ (0.01 mol/L) suspension.

#### Measurements of photosynthetic and growth variables of walnut saplings

The height and basal diameter of the walnut saplings were measured on 18 October 2016. Basal diameter was measured at 3 cm above the ground with a vernier caliper. To determine a tree’s crown diameter, we measured its crown extensions north, south, east and west with a tape.

A portable photosynthesis system (LI-6400; LI-COR, Inc., Lincoln, USA), with a red/blue light-emitting diode (LED) light source (LI6400-02B) mounted on a 6-cm^2^ clamp-on leaf chamber, was used to determine the photosynthesis rate under sunny and windless weather conditions. The net CO_2_ assimilation rate (Pn) and transpiration rate were measured on fully expanded walnut sapling leaves at similar development stages with a portable open-flow gas exchange system (LI-6400, LI-COR Inc., USA) during the late morning (9:00–11:00 h), with three duplications per treatment. Pn was measured one time along the season on 19 October 2016. In all cases, the air relative humidity, CO_2_ concentration and photon flux density were maintained at 60%–70%, 380 mmol·mol^−1^ and 800 mmol·m^−2^·s^−1^, respectively.

#### Principal component analysis (PCA)

PCA is a useful statistical technique in ecology which uses the covariance between variables in datasets to arrive at a linear representation of the system by orthogonal vectors, and it is a powerful tool that can identify correlations and qualities in the datasets^48^.

After the growing season (from June to October), the following six walnut saplings and soil properties were recorded: tree height, net photosynthesis rate, crown width, soil pH, soil moisture content and leaf transpiration rate. The fruit yield of walnut saplings was not measured since this study focused on the sapling stage of development. With these six parameters selected, it was possible to describe the key growth and physiological characteristics and soil properties of the walnut saplings. PCA allows us to reduce the relevant factors, which simplifies the analysis because the Box–Behnken analysis can be done with two response variables instead of six^49,50^.

Variance maximization rotation is a method of the Kaiser Normalization used in PCA: two principal components are chosen according to their highest initial values, then named Component 1 and Component 2. In this way, the six comprehensive parameters would be integrated into two components. The PCA results were incorporated into the Box–Behnken design by using Design-Expert software (v8.0.5, Stat-Ease, Minneapolis, USA)^51^.

#### Optimal levels of straw mulching by using the desirability function

The numerical optimization feature in Design-Expert software was used to obtain quality (treatments of pure rice straw, pure rapeseed straw, and mixture of rice and rapeseed straw), placement variations (treatments of straw mulching placement, beginning at the tree trunk outward to the mean radius of crown width, to one and half mean radius of crown width, as well as to the whole quadrat) and quantity (treatments of 3, 6 and 9 kg/m^2^) of straw mulching for the walnut saplings (Table 4). We set the threshold to achieve the maximum desirability parameters (close to 1) within the experimental range^52^. An optimal solution having the maximum desirability was selected; as part of the follow-up validation, studies were done to further confirm the process conditions.

#### Statistical analysis

A BBD for RSM with three factors and three levels was used to study the correlation between the combined effects of individual processes on both responses from two levels (Table 1). The experimental design matrix and the responses based on the experimental runs proposed by BBD are summarised in Table 2. No transformation was made to the dataset before analysing both the response variables. Between the linear, two-factor interaction (2FI) quadratic and cubic polynomials, the quadratic model type was suggested as the most suitable for this process by Design-Expert software, since it showed a lower standard deviation along with higher R^2^-values.

The significance of all polynomial terms was judged statistically by computing their associated F-values and P-values with an alpha probability of 0.01 and 0.05. The regression coefficients were then used to generate the response plots. To judge the adequacy of the equations, we examined their lack of fit as indicated by their R^2^-values. Specifically, the R^2^ of an equation refers to the proportion of variation in the response attributed to the model rather than random error. An R^2^ of 0.80 or greater indicates a good fit^53^. After selecting the best-fitting model on this basis, ANOVA was used to study the statistical significance of the regression coefficients^34^. For a given model parameter, when the associated F-value is larger, the P-value is smaller, and the corresponding coefficient is higher, the better fitting is the model^54^. The ANOVA analysis shown in Table 2 is presented to justify the significance and adequacy of the model, with the value of (Prob>F) used to determine the statistical significance of each combination.

#### Data availability statement

Data for all empirical growth parameters (tree height of walnut, net photosynthesis rate of walnut leaves, crown width, soil pH, soil moisture content, transpiration rate of walnut leaves) are included in this article in the results section and as supplementary material.

## Conclusion

Statistical interpretation (i.e., based on the F-value, R^2^, CV, adequacy of precision) revealed that the relevant parameters of the model were significant. The maximum THW (3.616 m) and NPR values (6.806 μmol·m^−2^·s^−1^) were found at the optimum process conditions; i.e., high quality (mixed quality) (0.915), high placement (all n) (0.978) and low quantity (3 kg/m^2^) (−0.904), by using RSM that involved independent parameters. These values were then further validated, by actually performing the experiment using the optimized values, and found to agree satisfactorily with the values predicted by our models. Overall, applying the appropriate quality, placement and quantity of straw mulching could contribute to an optimal high THW and NPR, and perhaps also to high potential yields of walnut. Although optimal conditions may differ from those used in any future practical applications, our results may be useful as a reference base for more research on straw mulching and its use in walnut orchards.

## Electronic supplementary material

Supplementary Information
