# Supplementary material for: Optimization of growth and production parameters of walnut (Juglans regia) saplings with response surface methodology
Source: Sci Rep. 2018 Jul 3;8:9992. doi: 10.1038/s41598-018-28345-6 (PMC6030184; doi:10.1038/s41598-018-28345-6)
Supplement: Supplementary file 1 — Supplementary Information [file 41598_2018_28345_MOESM1_ESM.docx]

| Run order | *Independent variables* | | | | *Dependent variables* | | |  |
| --- | --- | --- | --- | --- | --- | --- | --- | --- |
|  | Quality(X_1_) | Placement(X_2_) | Quantity(X_3_) | Soil NH_4_^+^-N(mg/kg) | | Soil NO_3_^-^-N(mg/kg) |  | |
| 1 | 0 | 1 | 1 | 4.96 | | 48.3 |  |  |
| 2 | 1 | 1 | 0 | 3.61 | | 73.84 |  |  |
| 3 | 0 | 0 | 0 | 4.35 | | 56.22 |  |  |
| 4 | 0 | -1 | 1 | 4.53 | | 56.36 |  |  |
| 5 | -1 | 0 | 1 | 3.58 | | 47.81 |  |  |
| 6 | -1 | -1 | 0 | 4.56 | | 58.55 |  |  |
| 7 | 0 | 0 | 0 | 4.35 | | 56.22 |  |  |
| 8 | -1 | 0 | -1 | 3.36 | | 38.35 |  |  |
| 9 | -1 | 1 | 0 | 4.7 | | 63.4 |  |  |
| 10 | 0 | 1 | -1 | 4.53 | | 58.71 |  |  |
| 11 | 0 | 0 | 0 | 4.35 | | 56.22 |  |  |
| 12 | 1 | 0 | -1 | 3.54 | | 66.76 |  |  |
| 13 | 0 | -1 | -1 | 4.44 | | 68.77 |  |  |
| 14 | 0 | 0 | 0 | 4.35 | | 56.22 |  |  |
| 15 | 1 | 0 | 1 | 3.54 | | 70.58 |  |  |
| 16 | 1 | -1 | 0 | 2.6 | | 45.55 |  |  |
| 17 | 0 | 0 | 0 | 4.35 | | 56.22 |  |  |

**Optimization of growth and production parameters of walnut** (***Juglans regia*) saplings with response surface methodology**

Dagang Song ^1,2^, Kaiwen Pan ^1*^, Aiping Zhang ^1,2^, Xiaogang Wu^1^, Akash Tariq^1,2^, Wenkai Chen ^1,2^, Zilong Li ^1,2^, Feng Sun ^1,2^, Xiaoming Sun ^1^, Olusanya Abiodun Olatunji^1,2^, Lin Zhang ^1^

**Supplementary materials**

S-Table 1 Soil mineral N properties in study site

The maximum THW (3.616 m) and NPR values (6.806μmol·m^-2^·s^-1^) were found at optimum process conditions; i.e., high quality (mixed quality) (0.915), high placement (all n) (0.978) and low quantity (3 kg/m^2^) (–0.904), by using RSM that involved independent parameters. We also found that the Soil NH_4_^+^-N (5.87mg/kg) and Soil NO_3_^-^-N (77.57mg/kg) highest than other combination (S-Table 1) at optimum process conditions; i.e., high quality (mixed quality) (0.915), high placement (all n) (0.978) and low quantity (3 kg/m^2^) (–0.904).Thus, we can explain the results according mineral N availability. Therefore, soil inorganic nitrogen also supports the best combination of straw mulching to promote the growth of walnutsaplings.

S-Table 2 Climate data of Langzhong

| Precipitation（mm） | 2012 | 2013 | 2014 | 2015 | 2016 |
| --- | --- | --- | --- | --- | --- |
| June  July  August  September  October  Mean | \| 104.6 \| \| --- \| \| 283.4 \| \| 213.0 \| \| 199.8 \| \| 67.7 \| \| 173.7 \| | \| 179.7 \| \| --- \| \| 432.0 \| \| 165.3 \| \| 163.2 \| \| 67.9 \| \| 201.62 \| | \| 69.0 \| \| --- \| \| 51.8 \| \| 116.7 \| \| 279.2 \| \| 142.8 \| \| 131.9 \| | \| 319.9 \| \| --- \| \| 81.4 \| \| 191.2 \| \| 259.8 \| \| 66.9 \| \| 183.8 \| | \| 36.7 \| \| --- \| \| 132.4 \| \| 50.8 \| \| 81.8 \| \| 50.0 \| \| 70.3 \| |

The above data are derived from the National Meteorological Station in Langzhong.





S-Figure1 Walnuts production of world and China during 2001-2012.
